# The Prognostic and Risk Factors for Children With High‐Risk Mature B‐Cell Non‐Hodgkin's Lymphoma: A Retrospective Multicenter Study

**DOI:** 10.1002/cam4.70309

**Published:** 2024-11-08

**Authors:** Xiaoming Wang, Luping Ding, Yongjun Fang, Jie Yan, Ju Gao, Liangchun Yang, Aiguo Liu, Jun Lu, Jingfu Wang, Aijun Zhang, Yijin Gao, Xiuli Ju

**Affiliations:** ^1^ Department of Pediatrics Qilu Hospital of Shandong University Jinan Shandong Province China; ^2^ Department of Hematology/Oncology Children's Hospital of Nanjing Medical University Nanjing Jiangsu Province China; ^3^ Department of Pediatric Oncology Tianjin Medical University Cancer Institute and Hospital Tianjin China; ^4^ Department of Pediatrics West China University Second Hospital of Sichuan University Chengdu Sichuan Province China; ^5^ Department of Pediatric Hematology/Oncology Xiangya Hospital of Central South University Changsha Hunan Province China; ^6^ Department of Pediatric Hematology/Oncology Tongji Hospital Affiliated to Tongji Medical College of Huazhong University of Science and Technology Wuhan Hubei Province China; ^7^ Department of Hematology/Oncology Children's Hospital of Soochow University Soochow Jiangsu Province China; ^8^ Department of Pediatric Oncology Shandong Cancer Hospital and Institute, Shandong First Medical University and Shandong Academy of Medical Sciences Jinan Shandong Province China; ^9^ Department of Hematology/Oncology School of Medicine, Shanghai Children's Medical Center, Shanghai Jiaotong University Shanghai China

**Keywords:** children, high‐risk mature B‐NHL, risk factors, rituximab

## Abstract

**Backgrounds and Aims:**

Our previous study (CCCG‐BNHL‐2015) reported the treatment strategies and outcomes of pediatric B‐cell non‐Hodgkin's lymphoma (B‐NHL) in China which showed that children in low‐risk groups already have a dramatically favorable prognosis. However, for high‐risk groups, the prognosis still needs to be improved. In this study, we aimed to identify the factors influencing prognosis in high‐risk groups (stage III and stage IV).

**Results:**

Our results revealed that gender, lactate dehydrogenase (LDH) level, stage at the time of diagnosis, and early complete remission (CR) achievement were significant factors influencing prognosis (*p* < 0.05). The 3‐year EFS rate for R4 group patients without rituximab treatment was only 25.0% ± 20.4%. Among all patients in stage IV, the 5‐year EFS rates for those with involvement of only bone marrow (BM) or central nervous system (CNS) were 83.0% ± 4.5%, 81.8% ± 8.2%, but the 5‐year EFS rates for those with both BM and CNS involved were only 37.5% ± 15.3% (*p* = 0.002). For stage III patients with LDH ≥ 4N, the 5‐year EFS rates for those achieving CR and those not achieving CR after 2 treatment cycle were 88.9% ± 5.2% and 67.9% ± 7.3%(*p* = 0.036).

**Conclusions:**

Therefore, R4 group patients benefited from rituximab treatment. However, children at stage III, LDH ≥ 4N not achieving CR after the 2nd treatment cycle, and those with both BM and CNS involved are still at a very high risk of treatment failure. This study serves as a crucial reference for optimizing risk stratification, refining treatment categorizations, and optimizing treatment protocols.

## 1 Introduction

Lymphoma ranks as the third most common cancer in children and adolescents, following leukemia and central nervous system (CNS) tumors. Among newly diagnosed lymphoma patients, mature B‐cell non‐Hodgkin's lymphoma (B‐NHL) constitutes approximately 50%–60% of cases [1, 2]. At present, the standard treatment approach for pediatric B‐NHL involves a combination of rituximab and chemotherapy, with risk stratification guiding the treatment decisions [3]. It is noticeable that children with low‐risk mature B‐NHL already have a dramatically favorable prognosis: nearly 100% as our previous study (CCCG‐BNHL‐2015) reported [4]. The addition of rituximab to standard chemotherapy could significantly improve EFS rates in pediatric and adolescent patients with high‐risk mature B‐NHL [5, 6, 7, 8]. Our previous study showed that the 4‐year EFS rate was improved with the addition of 4 doses of rituximab compared with the previous CCCG‐BNHL‐2010 study. However, there is still a subset of patients within high‐risk groups who exhibited refractory responses and experienced relapses. Additionally, managing relapsing or progressive lymphoma poses noticeable challenges in the treatment process. This multicenter, cohort, retrospective study aimed to optimize the risk stratification and improve the prognosis by analyzing the prevalence, clinical patterns, and factors influencing outcomes of Chinese children with high‐risk mature B‐NHL.

## 2 Patients and Methods

### 2.1 Patients

A total of 9 Chinese hospitals participated in this multicenter, retrospective study, including Shanghai Children's Medical Center of Shanghai Jiaotong University, Nanjing Children's Hospital of Nanjing Medical University, Tianjin Medical University Cancer Institute and Hospital, West China Second University Hospital of Sichuan University, Xiangya Hospital of Central South University, Tongji Hospital Affiliated to Tongji Medical College of Huazhong University of Science and Technology, Qilu Hospital of Shandong University, Children's Hospital of Soochow University, and Tumor Hospital of Shandong Frist Medical University. From March 2014 to June 2022, 435 patients, aged ≤ 18 years old with newly diagnosed high‐risk mature B‐NHL, were admitted to the above‐mentioned hospitals and involved in this study. They underwent regular chemotherapy and follow‐up according to the CCCG‐BNHL‐2015 protocol. The study was approved by the Ethics Committee of Shanghai Children's Medical Center (Shanghai, China; Approval No. NCT02405676), and written informed consent was obtained from all patients or their legal guardians.

### 2.2 Data Collection

The clinical data, including gender, age at the time of diagnosis, primary site, involved site, pathological type, serum lactate dehydrogenase (LDH) level, stage at the time of diagnosis, theoretical grouping, treatment grouping, the utilization of rituximab, c‐Myc, remission time, the time of the first event, follow‐up time, and final state were collected through the medical record system of each hospital. C‐Myc was tested according to immunohistochemistry or fluorescence in situ hybridization (FISH).

### 2.3 Diagnosis and Treatment

The diagnosis of B‐NHL was performed by tumor biopsy, cytological, immunological, and genetic examinations, excluding other types of hematological diseases. Pathologic classification was performed according to the 2008 WHO classification of lymphoid tissue tumors [9], and non‐Hodgkin lymphoma was clinically staged according to the St. Jude staging system for pediatric non‐Hodgkin lymphoma [10]. According to the results of immunohistochemistry, flow cytometry, bone marrow cytology, and cerebrospinal fluid cytology performed on tumor tissue, bone marrow, body fluid, and cerebrospinal fluid samples, patients in this study were classified based on the Murphy staging system. The high‐risk groups were summarized as follows: stage III included primary gastrointestinal masses, typically in the ileocecal region, with ascites or extension to adjacent organs. This stage encompassed lesions on both sides of the diaphragm, incorporating primary thoracic lesions (mediastinum, hilum, lung, pleura, or thymus), extensively and incompletely resected abdominal lesions, as well as paravertebral or epidural tumors with involvement of two or more extranodal masses, which may consist of involvement of two or more instances of bone and involvement of two or more instances of skin. Additionally, a single bone lesion accompanied by extranodal and/or non‐regional lymph node involvement was classified as stage III. Stage IV was characterized by CNS involvement, bone marrow infiltration, or both [5]. Definition of CNS involvement: presence of any of the following: (1) centrifugal detection of lymphoma cells in cerebrospinal fluid specimens; (2) the patient has clear symptoms and/or signs of central nervous system involvement, such as cranial nerve paralysis, which cannot be explained by other causes; (3) spinal cord infiltration; (4) isolated intracerebral tumor space‐occupying lesions. Definition of BM involvement: (1) tumor cells (usually ≥ 5% lymphoid blasts) on bone marrow smear; (2) or bone marrow biopsy showing focal tumor cell infiltration. The biological examination included measurement of LDH level. The LDH level was categorized into three groups: less than two times the normal value (< 2 N), two to four times the normal value (2 N‐4 N), and greater than and equal to four times the normal value (≥ 4 N). The high‐risk group included those who were diagnosed at stage III with an elevated LDH level or stage IV. Based on the stage at the time of diagnosis and LDH level, patients were stratified into R3 and R4 groups as follows: R3: patients in stage III and LDH < 4N; R4: patients in stage III and LDH ≥ 4N; or patients in stage IV; or patients with Burkitt‐type acute lymphoblastic leukemia (B‐AL). The treatment regimen, which was following the CCCG‐BNHL‐2015 protocol, was summarized as follows: R3: treatment sequence PA → BB → AA→BB → AA→BB; R4: treatment sequence PA → R‐BB → R‐AA→R‐BB → R‐AA→BB (Figure S1).

### 2.4 Evaluation of Treatment Effects

The therapeutic efficacy was evaluated after the 2nd cycle of treatment as outlined in the treatment protocol. The following criteria defined complete remission (CR): (1) disappearance of all lesions except for those in the bone, including the absence of tumor cells in the bone marrow and cerebrospinal fluid; (2) pathologically confirmed no residual lesions with tumor cells after surgical removal; (3) residual lesions less than 2 cm in size and inoperable by the surgeon; (4) no metabolic activity indicated by positron emission tomography‐computed tomography (PET‐CT) scan; (5) only one lesion less than 1 cm involving the liver or kidneys, which remained stable for more than 3 months. Partial remission (PR) was defined as a reduction of more than 25% in the two largest diameters of the tumor. The following criteria identified disease progression or recurrence: (1) reduction of 25% or less in the maximum diameter of the tumor; (2) appearance of new localized lesions, reappearance of naive cells in the cerebrospinal fluid, or more than 5% naive cells in the bone marrow, as confirmed by MYC fluorescence in situ hybridization (FISH) testing; (3) enlargement in the area or size of the original lesion. In the present study, we focused on assessing whether the children achieved early complete remission after the 2nd cycle of treatment.

### 2.5 Follow‐Up

The endpoint of follow‐up was March 2023, and the median follow‐up time was 37 (range, 0.1–98.3) months. EFS was defined as the time from the start of treatment to the occurrence of any event. Events included a range of outcomes, such as non‐remission, recurrence, death, development of a second tumor, abandonment of treatment, and other pertinent factors.

### 2.6 Statistical Analysis

Statistical analysis was performed using SPSS 26.0 software (IBM, Armonk, NY, USA). The normally distributed measurement data were expressed as mean ± standard deviation (X¯ ± s), the abnormally distributed measurement data were presented as mean (Q1, Q3), and the count data were expressed as percentage (%). Kaplan–Meier method was employed for survival analysis. The log‐rank test was utilized to compare the survival rates between the groups. *p* < 0.05 was considered statistically significant.

## 3 Results

### 3.1 The Clinical Characteristics of Children in the High‐Risk Group

The study included 435 eligible high‐risk patients. Table 1 summarizes patients' baseline characteristics. According to the theoretical grouping, there were 233 (53.6%) and 202 (46.4%) patients in the R3 and R4 groups, respectively. The incidence rate was significantly higher in male patients than that in female patients (80.7% vs. 19.3%). The median age of diagnosis was 80.5 (7.7–194.7) months. Out of the total patient cohort, 318 (73.1%) patients were diagnosed with BL and 64 (14.7%) patients with diffuse large B‐cell lymphoma (DLBCL). Moreover, 286 (65.7%) patients were found to be c‐Myc positive (+) by FISH or immunohistochemistry. The most common primary site was the abdomen, accounting for about 58.2%. Furthermore, in terms of LDH level, 218 (50.1%) patients had LDH < 2N, 70 (16.1%) patients had LDH 2–4N, and 147 (33.8%) patients had LDH ≥ 4N. The distribution of stages in the patient cohort was as follows: 310 (71.3%) patients in stage III, and 125 (28.7%) patients in stage IV. According to the use of rituximab, there were 246 (56.6%) and 189 (43.4%) cases in the rituximab + chemotherapy (RTX + chemotherapy) and chemotherapy groups, respectively. According to the involvement of BM and CNS diseases, there were 89 (20.5%) cases of BM‐only involvement, 23 (5.3%) cases of CNS‐only involvement, and 13 (3.0%) cases of BM + CNS involvement. The therapeutic efficacy was assessed after the 2nd cycle of treatment, including 269 (61.8%) patients with CR, 164 (37.7%) patients with incomplete remission, and 2 (0.5%) patients who were not assessed. Table S1 summarizes patients' baseline characteristics who experienced events.

**TABLE 1:** Baseline characteristics of all patients.

	R3 (233)	R4 (202)	Total (435)
Gender			
Male	190 (81.5%)	161 (79.7%)	351 (80.7%)
Female	43 (19.5%)	41 (20.3%)	84 (19.3%)
Age (month, median)	9.5–183.7 (86.6)	7.7–194.7 (73.7)	7.7–194.7 (80.5)
LDH			
< 2 N	188 (80.7%)	30 (14.9%)	218 (50.1%)
2 N‐4 N	45 (19.3%)	25 (12.4%)	70 (16.1%)
≥ 4 N	0 (0)	147 (72.7%)	147 (33.8%)
Stage			
III	233 (100%)	77 (38.1%)	310 (71.3%)
IV	0 (0)	125 (61.9%)	125 (28.7%)
Pathological diagnosis			
Burkitt	172 (73.8%)	146 (72.3%)	318 (73.1%)
DLBCL	43 (18.5%)	21 (10.4%)	64 (14.7%)
High‐grade B‐cell lymphoma	12 (5.2%)	6 (3.0%)	18 (4.1%)
Mature B‐cell leukemia	0 (0)	16 (7.9%)	16 (3.7%)
other types	6 (2.5%)	13 (6.4%)	19 (4.4%)
C‐Myc			
Positive	141 (60.5%)	145 (71.8%)	286 (65.7%)
Negative	55 (23.6%)	37 (18.3%)	92 (21.2%)
No check	37 (15.9%)	20 (9.9%)	57 (13.1%)
The treatment of rituximab			
RTX + chemotherapy	57 (24.5%)	189 (93.6%)	246 (56.6%)
Chemotherapy	176 (75.5%)	13 (6.4%)	189 (43.4%)
Initial sites			
Head and neck	70 (30.5%)	53 (26.2%)	123 (28.3%)
Thorax	13 (5.5%)	16 (7.9%)	29 (6.7%)
Abdomen	147 (63.1%)	106 (52.5%)	253 (58.2%)
Involvement			
Only BM	—	89 (44.1%)	89 (20.5%)
Only CNS	—	23 (11.4%)	23 (5.3%)
BM + CNS	—	13 (6.4%)	13 (3.0%)
Evaluation after the 2nd cycle of treatment			
Complete remission (CR)	160 (68.7%)	109 (54.0%)	269 (61.8%)
No CR	71 (30.5%)	93 (46.0%)	164 (37.7%)
Not evaluation	2 (0.8%)	—	2 (0.5%)

### 3.2 Prognostic Factors for High‐Risk Children With Mature B‐NHL

**FIGURE 1:**
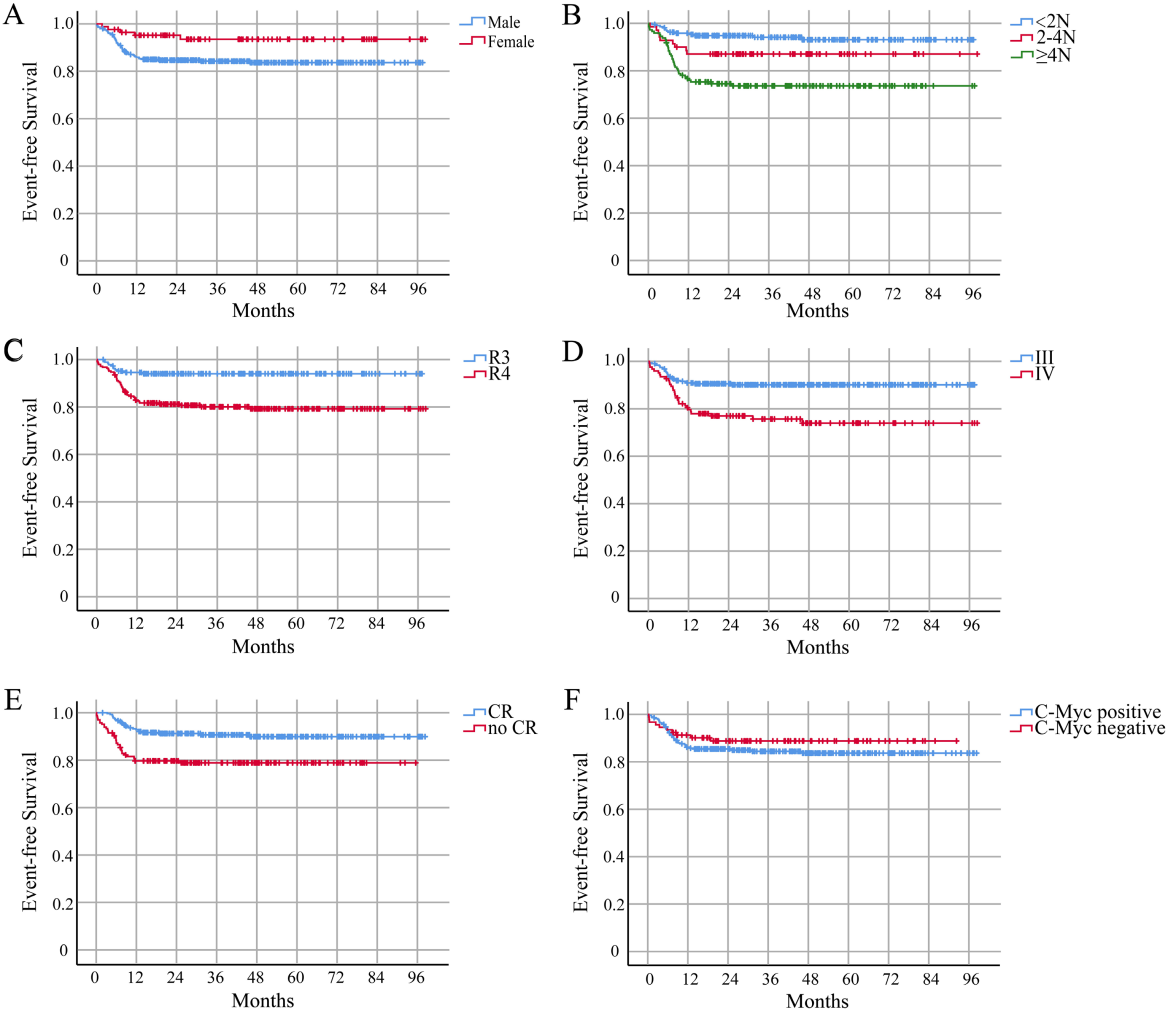
Survival curves of all patients. (A) The EFS curves for gender. (B) The EFS curves for LDH < 2 N, 2 N‐4 N, and ≥ 4 N. (C) The EFS curves for the treatment group. (D) The EFS curves for Stage III and IV. (E) The EFS curves for achieving CR or no CR after the 2nd cycle of treatment. (F) The EFS curves for c‐Myc positive or c‐Myc negative.

The median follow‐up time for 435 patients was 37 (range, 0.1–98.3) months. The 1‐, 3‐, and 5‐year EFS rates in the high‐risk group were 87.8% ± 1.6%, 86.0% ± 1.7%, and 85.6% ± 1.8%, respectively. The 5‐year EFS rates for male and female patients were 83.6% ± 2.1% and 93.5% ± 2.9%, respectively (*p* = 0.024) (Figure 1A). Patients with a lower LDH level had significantly longer survival (*p* < 0.001). The 5‐year EFS rates for LDH < 2N, 2N‐4N, and ≥ 4 N were 93.1% ± 1.9%, 87.1% ± 4.0%, and 73.7% ± 3.7%, respectively (*p* < 0.001) (Figure 1B). The 5‐year EFS rates for the R3 and R4 groups were 94.0% ± 1.8% and 79.2% ± 2.7%, respectively (*p* < 0.001) (Figure 1C). The 5‐year EFS rates for stages III and IV were 90.1% ± 1.7% and 74.0% ± 4.3%, respectively (*p* < 0.001) (Figure 1D). The 5‐year EFS rates for patients achieving CR and no CR after 2 cycles of treatment were 89.9% ± 2.0% and 78.8% ± 3.2%, respectively (*p* < 0.001) (Figure 1E). The 5‐year EFS rates for c‐Myc‐positive group and c‐Myc‐negative group were 83.7% ± 2.3% and 88.9% ± 3.4%, respectively (*p* = 0.311) (Figure 1F).

In the present study, univariate COX regression analysis was performed with consideration of events as dependent variables and clinical characteristics as independent variables (Table 2). The results exhibited no significant correlation among age at the time of diagnosis, pathological type, the use of rituximab, c‐Myc, and prognosis. The indicators with clinical significance (*p* < 0.05) in the univariate analysis were utilized as independent variables, and the univariate factors that interfered with each other were excluded (Table 3). The results revealed that gender, LDH level, stage at the time of diagnosis, and CR achievement after the 2nd cycle of treatment were significant factors influencing prognosis (*p* < 0.05).

**TABLE 2:** Single factor analysis of different risk factors.

	*B*	SE	Wald	*p*	Exp (*B*)	95.0% CI for Exp (*B*)	
						Lower	Upper
Gender	1.011	0.467	4.686	0.030	2.749	1.100	6.867
Diagnostic age	0.001	0.003	0.026	0.872	1.001	0.994	1.007
LDH	0.777	0.157	24.567	< 0.001	2.176	1.600	2.959
Pathological diagnosis	−0.011	0.125	0.008	0.930	0.989	0.774	1.264
Stage	0.973	0.258	14.183	< 0.001	2.645	1.594	4.388
Treatment group	1.257	0.334	14.193	< 0.001	3.515	1.828	6.760
The treatment of Rituximab	0.496	0.278	3.187	0.074	1.641	0.953	2.828
CR (after the 2nd cycle of treatment)	0.906	0.264	11.812	0.001	2.474	1.476	4.147
C‐Myc	−0.353	0.350	1.016	0.314	0.703	0.354	1.396

**TABLE 3:** Multivariate analysis of different risk factors.

	*B*	SE	Wald	*p*	Exp (*B*)	95.0% CI for Exp (*B*)	
						Lower	Upper
Gender	1.063	0.468	5.163	0.023	2.896	1.157	7.245
LDH	0.661	0.168	15.542	< 0.001	1.937	1.395	2.692
Stage	0.628	0.271	5.352	0.021	1.873	1.101	3.188
CR (After the 2nd cycle of treatment)	0.798	0.264	9.106	0.003	2.221	1.323	3.730

### 3.3 Efficacy and Safety of the Combination of Rituximab

**FIGURE 2:**
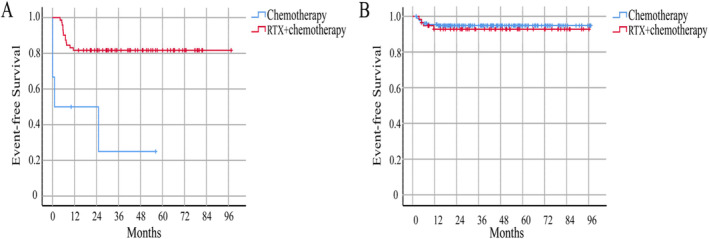
EFS curves of stage III. (A) The EFS curves for RTX+ chemotherapy group and chemotherapy group of stage III and LDH ≥ 4 N patients. (B) The EFS curves for RTX+ chemotherapy group and chemotherapy group of stage III and LDH < 4 N patients.

For patients receiving rituximab in stage III and LDH ≥ 4N, the 5‐year EFS rate for the RTX + chemotherapy group was 81.7% ± 4.6%, and the 3‐year EFS rate for the chemotherapy group was only 25.0% ± 20.4% (*p* < 0.001) (Figure 2A). The 5‐year EFS rate for stage IV patients receiving rituximab was 74.0% ± 4.3% (*p* < 0.001), which was improved compared with CCCG‐BNHL‐2010 (Figure 1D). Despite an increase of the included case numbers, the 5‐year EFS rates for RTX + chemotherapy group and chemotherapy group were 92.8% ± 3.5% and 94.9% ± 1.6% in R3 group, respectively, which exhibited no statistically significant difference as previous study reported (*p* = 0.585) (Figure 2B).

Table 4 lists the events that occurred in all patients according to the grouping and utilization of rituximab. Among all 435 patients, 60 (13.8%) events occurred, including 43 (9.9%) progression/recurrence events, 11 (2.5%) treatment‐related deaths, 2 (0.5%) tumor lysis‐related deaths, 1 death of second cancer (0.2%), and 3 deaths of unknown cause (0.6%). In the subset of patients belonging to the R4 group who did not receive rituximab, 10 out of 13 (76.9%) patients experienced mortality. All 11 treatment‐related deaths were caused by infections, in which the RTX+ chemotherapy group accounted for 3 (1.2%) and the chemotherapy group accounted for 8 (4.1%), respectively. According to the incidence of treatment‐related death in the R3 group, there were 1 (1.8%) and 4 (2.2%) cases in the RTX + chemotherapy and chemotherapy groups, respectively (*p* = 1.000). Of the 60 events, 54 (90.0%) occurred within the first year of diagnosis. Notably, all events in the R3 group occurred within the first year. Hence, the importance of evaluation and follow‐up within a 1‐year timeframe cannot be overstated.

**TABLE 4:** Advent events.

	R3 (233)		R4 (202)	
	RTX+ chemotherapy (57)	Chemotherapy (176)	RTX+ chemotherapy (189)	Chemotherapy (13)
Total number of events	4	9	37	10
Total number of deaths	3	7	32	10
Relapse/progression	3^a^	5^b^	32^c^	3
Treatment‐related deaths^d^	1	4	2	4
Unknown cause deaths	0	0	2	1
Tumor lysis deaths	0	0	0	2
Second cancer deaths	0	0	1	0

^a^ 2 patients died, 1 patient survived after CART.

^b^ 3 patients died, 2 patients survived after CART.

^c^ 27 patients died, 2 patients survived after CART, and 1 patient survived after autologous stem cell transplantation.

^d^ All treatment‐related deaths were caused by infections.

### 3.4 Identification of Patients Who Were at a Very High Risk of Treatment Failure

**FIGURE 3:**
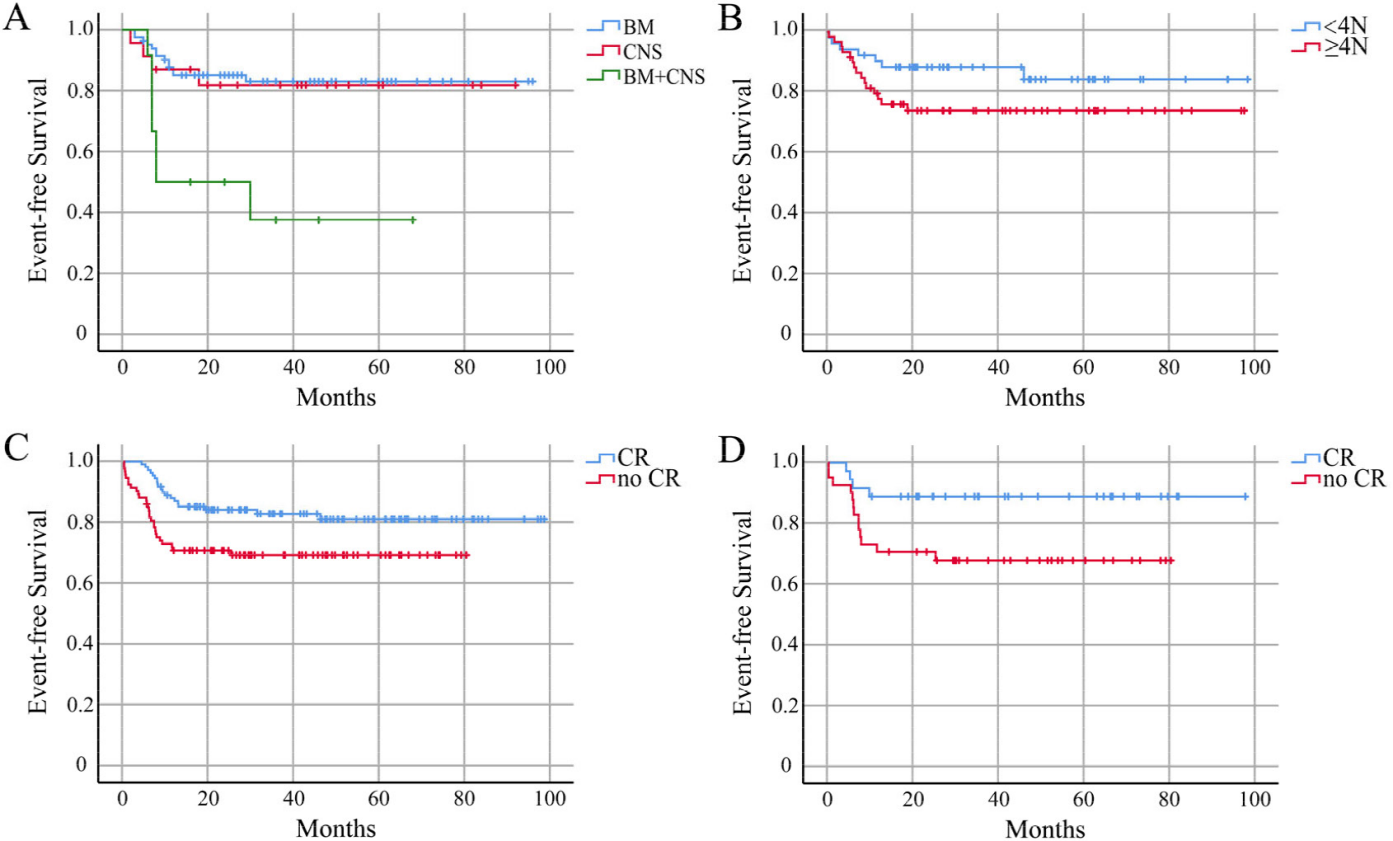
EFS curves of R4. (A) The EFS curves for the involvement of BM‐only, CNS‐only, and BM + CNS of all stage IV patients. (B) The EFS curves for LDH < 4 N or ≥ 4 N of patients with BM/CNS‐only involvement. (C) The EFS curves for achieving CR or no CR after the 2nd cycle of treatment of R4 patients. (D) The EFS curves for achieving CR or no CR after the 2nd cycle of treatment of stage III and LDH ≥ 4 N patients.

For patients receiving rituximab in R4 patients, the 5‐year EFS rate for the RTX + chemotherapy group was 80.4% ± 4.5%. The combination of rituximab improved the overall survival of children with high‐risk mature B‐NHL, but some children still cannot benefit. We further analyzed the clinical characteristics of children with poor prognosis in the high‐risk group. For stage IV patients receiving rituximab, the 5‐year EFS rates for the involvement of BM‐only, CNS‐only, and BM + CNS were 83.0% ± 4.5%, 81.8% ± 8.2%, and 37.5% ± 15.3%, respectively (*p* = 0.002) (Figure 3A). The 5‐year EFS rates for patients’ involvement of BM/CNS‐only with LDH < 4N or ≥ 4N were 84.4% ± 5.8% and 74.1% ± 5.8%, respectively (*p* = 0.114) (Figure 3B).

In the analysis of all patients in the R4 group, the 5‐year EFS rates for patients achieving CR and no CR after the 2nd cycle of treatment were 81.1% ± 4.0% and 69.3% ± 4.9%, respectively (*p* < 0.001) (Figure 3C). Interestingly, stage IV patients indicated that the 5‐year EFS rates for achieving CR and no CR after the 2nd cycle of treatment were 76.7% ± 5.4% and 70.3% ± 6.4%, respectively (*p* = 0.140) (Figure S2). This information reminded us that the early treatment response showed more significance in predicting the prognosis of stage III patients. As expected, for stage III patients with LDH ≥ 4N, the 5‐year EFS rates for achieving CR and no CR after the 2nd cycle of treatment were 88.9% ± 5.2% and 67.9% ± 7.3%, respectively (*p* = 0.036) (Figure 3D). But for stage III patients with LDH < 4N, the 5‐year EFS rates for achieving CR and no CR after the 2nd cycle of treatment were 96.2% ± 1.5% and 91.4% ± 3.4%, respectively (*p* = 0.136). Therefore, patients with involvement of both BM and CNS, involvement of BM/CNS‐only but LDH ≥ 4N, and those stage III patients with LDH ≥ 4N who can achieve no CR after the 2nd cycle of treatment are at a very high risk of treatment failure. Table 5 presents the EFS rates for stage III and IV patients.

**TABLE 5:** The EFS of stages III and IV.

	Number of patients	Number of events	The 5‐year EFS	*p*
Stage III				< 0.001
< 4 N, CR after the 2nd cycle of treatment	160	6	96.2% ± 1.5%	0.136
< 4 N, no CR after the 2nd cycle of treatment	71	6	91.4% ± 3.4%	
≥ 4 N, CR after the 2nd cycle of treatment	36	4	88.9% ± 5.2%	0.036
≥ 4 N, no CR after the 2nd cycle of treatment	41	13	67.9% ± 7.3%	
Stage IV				0.001
BM‐only	89	18	78.5% ± 4.5%	
CNS‐only	23	4	81.8% ± 8.2%	
BM/CNS‐only, LDH < 4 N	52	7	84.4% ± 5.8%	0.114
BM/CNS‐only, LDH ≥ 4 N	60	15	74.1% ± 5.8%	
BM + CNS	13	8	34.7% ± 14.4%	

## 4 Discussion

In the present study, the clinical characteristics, outcomes, and prognostic factors of ‘real‐world’ children with high‐risk mature B‐NHL were assessed in China. The mature B‐NHL encompasses various pathological types, including BL, DLBCL, and high‐grade B‐cell lymphoma [11]. Among them, BL is the most prevalent type in pediatric B‐NHL cases [12]. In the present study, the 1‐, 3‐, and 5‐year EFS rates in the high‐risk group were 87.8% ± 1.6%, 86.0% ± 1.7%, and 85.6% ± 1.7%, respectively. The EFS rate was significantly higher than the 2‐year EFS rate of 76.0% reported in the CCCG‐BNHL‐2010 study [13]. However, some children in high‐risk groups still cannot benefit from the treatment options and show refractory and relapse.

LDH level is a noticeable indicator for assessing B‐NHL patients' prognosis. An elevated serum LDH level can indicate a worsening condition in patients. According to the CCCG‐BNHL‐2015 guidelines, the LDH level could be categorized as < 2N, 2N‐4N, and ≥ 4N. Numerous studies have demonstrated that the LDH level exceeding 2 times the normal value (LDH ≥ 2N) could be associated with a significantly poorer prognosis [14, 15, 16]. In the present study, it was revealed that the 5‐year EFS rates were 93.1% ± 1.9%, 87.1% ± 4.0%, and 73.7% ± 3.7% for LDH < 2N, 2N‐4N, and ≥ 4N, respectively (*p* < 0.001). It was noted that the survival rates worsened as the LDH level increased. These findings may be valuable for future research utilizing LDH levels, promoting the development of further precise treatment strategies. Furthermore, it was indicated that patients who achieved CR after the 2nd cycle of treatment had higher survival rates, highlighting that children who responded well to early treatment had an increased likelihood of long‐term survival. Consequently, attention should be given to adjusting the treatment approach for children who do not achieve CR after the 2nd cycle of treatment.

Standard treatments for mature B‐NHL, such as radiotherapy and chemotherapy, can lead to rapid initial responses, whereas the risk of relapse remains high [17]. In this context, rituximab, a chimeric monoclonal antibody that targets CD20, has emerged as an important therapeutic option [18]. At present, rituximab in combination with chemotherapy is the standard therapy for the majority of pediatric patients with mature B‐NHL [19]. Given the more favorable outcomes found in children with mature B‐cell lymphomas treated with conventional chemotherapy without rituximab compared with adult data, it was crucial to examine the specific influence of adding rituximab on pediatric patients. Evaluating the safety and efficacy of incorporating rituximab into therapeutic regimens for managing children with B‐NHL has been a central concentration of thorough investigation over the past decade. A comprehensive study has indicated a risk of prolonged hypogammaglobulinemia in children with high‐risk mature B‐NHL treated with rituximab chemotherapy, although serious infections are infrequent [20].

The strength of this study is its real‐world design, which minimized selection bias and allowed the depiction of the therapeutic and prognostic outcomes of children with high‐risk, mature B‐NHL in China. The 2‐year EFS rates for stages III and IV were 75.1% ± 5.4% and 52.5% ± 13.1% respectively in CCCG‐BNHL‐2010 [4]. In our study, the 5‐year EFS rates for stages III and IV were 90.1% ± 1.7% and 74.0% ± 4.3%, respectively in CCCG‐BNHL‐2015. Therefore, combination with rituximab can improve EFS in children with high‐risk, mature B‐NHL. This study also revealed that the use of rituximab did not increase the incidence of treatment‐related death and infection‐related death in the high‐risk group.

The combination of rituximab improved the overall survival of children with high‐risk mature B‐NHL, but some children still cannot benefit. We further analyzed the clinical characteristics of children with poor prognosis in the high‐risk group. Children with advanced mature B‐NHL mainly present with BM, CNS, or involvement of both, necessitating aggressive therapy [21, 22, 23]. It can be concluded that the inclusion of rituximab in a modified treatment regimen has significantly improved the treatment efficacy for Chinese children with CNS+ and BM+ B‐NHL. For stage IV patients receiving rituximab, the 5‐year EFS rates for the involvement of BM‐only, CNS‐only, and BM + CNS were 83.0% ± 4.5%, 81.8% ± 8.2%, and 37.5% ± 15.3%, respectively (*p* = 0.002). Importantly, it was found that patients with involvement of both BM and CNS had a significantly worse prognosis than those with involvement of BM‐only and CNS‐only. Despite the incorporation of rituximab into the therapy, their survival rates have remained notably low. The 5‐year EFS rates for patients’ involvement of BM/CNS‐only with LDH ≥ 4N and LDH < 4N were 84.4% ± 5.8% and 74.1% ± 5.8%, respectively (*p* = 0.114). This result showed no statistically significant might because the patient number was limited. Therefore, patients with involvement of both BM and CNS are at a very high risk of treatment failure.

In the analysis of all patients in the R4 group, the 5‐year EFS rates for patients achieving CR and no CR after the 2nd cycle of treatment were 81.1% ± 4.0% and 69.3% ± 4.9%, respectively (*p* < 0.001). The difference among stage IV patients was not significant which reminded us that the treatment response showed more significance in predicting the prognosis of stage III patients. As expected, for stage III patients with LDH ≥ 4N, the 5‐year EFS rates for achieving CR and no CR after the 2nd treatment cycle were 88.9% ± 5.2% and 67.9% ± 7.3%, respectively (*p* = 0.036). Therefore, those stage III patients with LDH ≥ 4N who can achieve no CR after the 2nd cycle of treatment could not benefit enough from the continuous treatment even with the addition of 4 doses of rituximab. This information provided valuable insights for further optimizing risk stratification to adjust these patients into extremely high‐risk groups.

Therefore, these extremely high‐risk patients cannot benefit enough from the current intensive frontline treatment approaches. It will be necessary to evaluate new drugs or alternative strategies for their treatment. Chimeric antigen receptor T cell (CART) therapy is a valuable new approach to treating patients with B‐cell malignancies [24], and it has been utilized to treat pediatric refractory or relapsed mature B‐NHL (r/r MB‐NHL) [25]. Autologous hematopoietic stem cell transplantation (ASCT) is also a good therapeutic option, which prolongs disease progression‐free survival and overall survival by reproducing established normal hematopoietic and immune function in patients [26]. In the present study, 5 patients were treated with CART, and one patient was treated with ASCT after progression/relapse, and they all achieved CR. Enhancing the treatment outcomes for these patients could involve considering alternative therapies at an early stage. Gene transfer therapies, such as CART therapy, and small molecule inhibitors targeting the B‐cell receptor signaling pathway, may provide potential benefits to these patients [27].

In a previous study, it was noted that all relapses occurred within a relatively early time frame of 2–4 months following the completion of treatment [1]. Similarly, the present study revealed that the majority of events, such as progression and recurrence, occurred within the first year after the initial diagnosis. With longer follow‐ups, the likelihood of such events decreased. Among 60 events, 54 (90.0%) events occurred within the first year after diagnosis. Specifically, all events in the R3 group, consisting of 238 patients, occurred within the first year following diagnosis. These findings underscore the importance of comprehensive assessment and follow‐up during the initial year following diagnosis, as they are pivotal in the early detection and effective management of potential events.

## 5 Conclusions

In conclusion, EFS rates were found to improve significantly in children with high‐risk, mature B‐NHL, with a 5‐year EFS rate of 85.6% ± 1.8% for all patients. The combination of rituximab improved the overall survival of children with high‐risk mature B‐NHL, but some children still cannot benefit. Survival differences were identified to be associated with gender, LDH level, stage at the time of diagnosis, and the status of achieving CR after the 2nd cycle of chemotherapy. We further analyzed the clinical characteristics of children with poor prognosis and recognized children with extremely high risk as follows: stage IV patients with involvement of both BM and CNS, involvement of BM/CNS‐only but LDH ≥ 4N, and those stage III patients with LDH ≥ 4N who can achieve no CR after the 2nd cycle of treatment are at a very high risk of treatment failure. Therefore, optimizing treatment regimens, such as CART therapy and autologous stem cell transplantation, should be contemplated at the earliest stages for those patients. The present study provided valuable insights for further optimizing risk stratification, refining treatment regimens, and implementing individualized and precise treatment approaches.

## Author Contributions

**Xiaoming Wang:** conceptualization (equal), formal analysis (equal), investigation (equal), visualization (equal), writing – review and editing (lead). **Luping Ding:** formal analysis (equal), methodology (equal), software (lead), writing – original draft (lead). **Yongjun Fang:** data curation (equal), resources (equal). **Jie Yan:** data curation (equal), resources (equal). **Ju Gao:** data curation (equal), resources (equal). **Liangchun Yang:** data curation (equal), resources (equal). **Aiguo Liu:** resources (equal), software (equal). **Jun Lu:** resources (equal), software (equal). **Jingfu Wang:** resources (equal). **Aijun Zhang:** conceptualization (equal), methodology (equal), project administration (equal), supervision (equal), validation (equal), visualization (equal). **Yijin Gao:** resources (equal), writing – review and editing (equal). **Xiuli Ju:** formal analysis (supporting), methodology (supporting), project administration (equal), supervision (equal), validation (equal).

## Ethics Statement

The study was approved by the Ethics Committee of Shanghai Children's Medical Center (Shanghai, China; Approval No. NCT02405676). All participants provided written informed consent before enrollment.

## Conflicts of Interest

The authors declare no conflicts of interest.

## Supporting information


Figure S1.

Table S2.

## Data Availability

The data that support the findings of this study are available from the corresponding author upon a reasonable request.
