# Supplementary material for: The Prognostic and Risk Factors for Children With High‐Risk Mature B‐Cell Non‐Hodgkin's Lymphoma: A Retrospective Multicenter Study
Source: Cancer Med. 2024 Nov 8;13(21):e70309. doi: 10.1002/cam4.70309 (PMC11544326; doi:10.1002/cam4.70309)
Supplement: Supplementary file 1 — Figure S1. [file CAM4-13-e70309-s002.docx]

**Figure S1: Treatment process.**

**

**

**Figure S2: The EFS curves for achieving CR or no CR after the 2nd cycle of treatment of stage IV patients.**

**
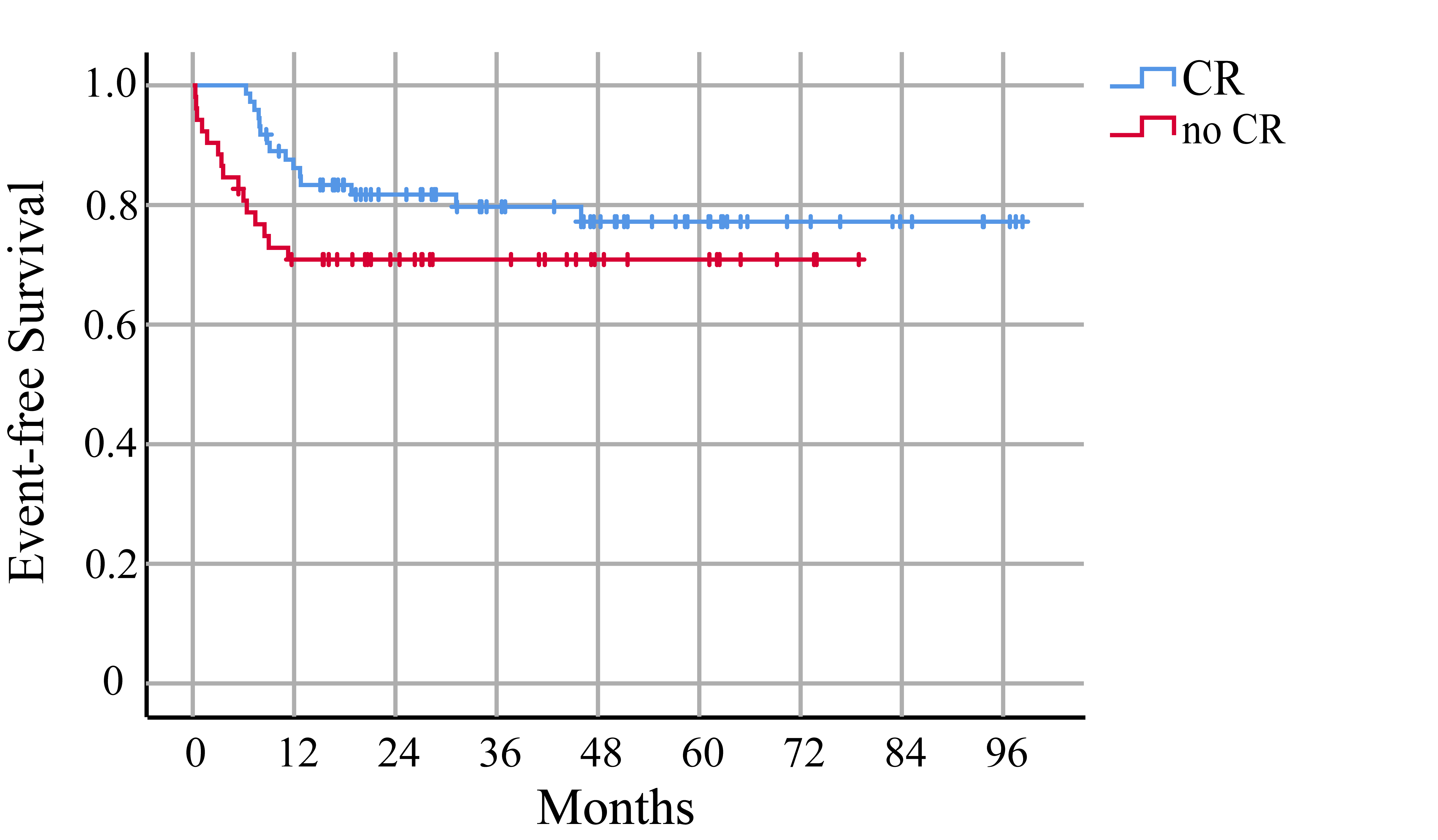
**
