# Supplementary material for: The Prognostic and Risk Factors for Children With High‐Risk Mature B‐Cell Non‐Hodgkin's Lymphoma: A Retrospective Multicenter Study
Source: Cancer Med. 2024 Nov 8;13(21):e70309. doi: 10.1002/cam4.70309 (PMC11544326; doi:10.1002/cam4.70309)
Supplement: Supplementary file 2 — Table S2. [file CAM4-13-e70309-s001.docx]

**Table S1: Baseline characteristics** **of patients who have events**

|  | **R3** (13) | **R4** (47) | **Total** (60) |
| --- | --- | --- | --- |
| **Gender** |  |  |  |
| Male | 12(92.3%) | 43(91.5%) | 55 (91.7%) |
| Female | 1(7.7%) | 4 (8.5%) | 5(8.3%) |
| **Age (month, median)** | 23.7-162.3(82.0) | 16.3-178.1(84.2) | 16.3-178.1(83.3) |
| **LDH** |  |  |  |
| ＜2N | 9(69.2%) | 4(8.5%) | 13(21.7%) |
| 2N-4N | 4(30.8%) | 5(10.6%) | 9(15.0%) |
| ≥4N | 0(0) | 38(80.9%) | 38(63.3%) |
| **Stage** |  |  |  |
| III | 13((100%) | 17(36.2%) | 30(50.0%) |
| IV | 0(0) | 30(63.8%) | 30(50.0%) |
| **Pathological diagnosis** |  |  |  |
| Burkitt | 10(76.9%) | 34(72.3%) | 44(73.3%) |
| DLBCL | 3(23.1%) | 3(6.4%) | 6(10.0%) |
| High-grade B-cell lymphoma | 0(0) | 2(4.3%) | 2(3.3%) |
| Mature B-cell leukemia | 0(0) | 7(14.9%) | 7(11.7%) |
| other types | 0(0) | 1(2.1%) | 1(1.7%) |
| **C-myc** |  |  |  |
| Positive | 9(69.2%) | 35(74.5%) | 44(73.3%) |
| Negative | 2(15.4%) | 8(17.0%) | 10(16.7%) |
| No check | 2(15.4%) | 4(8.5%) | 6(10.0%) |
| **The treatment of Rituximab** |  |  |  |
| RTX + chemotherapy | 4(30.8%) | 37(78.7%) | 41(68.3%) |
| Chemotherapy | 9(69.2%) | 10(21.3%) | 19(31.7%) |
| **Initial sites** |  |  |  |
| Head and neck | 3(23.1%) | 14(29.8%) | 17(28.3%) |
| Thorax | 0(0) | 3 (6.4%) | 3(5.0%) |
| Abdomen | 11(84.6%) | 25(53.2%) | 36(60.0%) |
| **Involvement** |  |  |  |
| Only BM | - | 18(38.3%) | 18(30.0%) |
| Only CNS | - | 4(8.5%) | 4(6.7%) |
| BM+CNS | - | 8(17.0%) | 8(13.3%) |
| **Evaluation after the 2nd cycle of treatment** | | | |
| Complete remission (CR) | 6(46.2%) | 19(40.4%) | 25(41.7%) |
| No CR | 6(46.2%) | 28(59.6%) | 34(56.6%) |
| Not evaluation | 1(7.6%) | - | 1(1.7%) |
